# Selective dopamine D2 receptor deletion from Nkx6.2 expressing cells causes impaired cognitive, motivation and anxiety phenotypes in mice

**DOI:** 10.1038/s41598-023-46954-8

**Published:** 2023-11-09

**Authors:** Lucila Bechelli, Eugenia Tomasella, Sofia Lopez Cardoso, Martina Belmonte, Diego M. Gelman

**Affiliations:** 1 Instituto de Biología y Medicina Experimental (IBYME). Fundación IBYME., Consejo Nacional de Investigaciones Científicas y Técnicas (CONICET)., Vuelta de Obligado 2490, C1428ADN Ciudad Autónoma de Buenos Aires, Argentina; 2 Universidad Argentina de la Empresa (UADE), Lima 757, C1073AAO Ciudad Autónoma de Buenos Aires, Argentina

**Keywords:** Development of the nervous system, Diseases of the nervous system, Learning and memory, Molecular neuroscience, Motivation, Neurogenesis, Neurodevelopmental disorders, Depression, Schizophrenia

## Abstract

Abnormal dopamine neurotransmission is a common trait of some psychiatric diseases, like schizophrenia or bipolar disorder. Excessive dopaminergic tone in subcortical brain regions is associated with psychotic episodes, while reduced prefrontal dopaminergic activity is associated with impaired cognitive performance and reduced motivation, among other symptoms. Inhibitory interneurons expressing the calcium binding protein parvalbumin are particularly affected in both schizophrenia and bipolar disorder, as they set a fine-tuned physiological inhibitory/excitatory balance. Parvalbumin and somatostatin interneuron subtypes, are born from the medial ganglionic eminence and require the sequential expression of specific transcription factors for their specification, such as Nkx6.2. Here, we aimed at characterizing in detail interneuron subtypes derived from Nkx6.2 expressing progenitors by the generation of an Nkx6.2 Cre transgenic mouse line. We show that Nkx6.2 specifies over a third part of the total population of cortical somatostatin interneurons, preferentially at early developmental time points, whereas at late developmental stages, Nkx6.2 expressing progenitors shift to parvalbumin interneuron specification. Dopamine D2 receptor deletion from Nkx6.2 expressing progenitors causes abnormal phenotypes restricted to cognitive, motivation and anxiety domains. Our results show that Nkx6.2 have the potential to specify both somatostatin and parvalbumin interneurons in an opposite timed program and that DRD2 expression is required in Nkx6.2 expressing progenitors to avoid impaired phenotypes commonly associated to the pathophysiology of psychiatric diseases.

## Introduction

Dopamine dysregulation is a hallmark of psychiatric diseases, like bipolar disorder or schizophrenia. The dopaminergic hypothesis of these diseases postulates that an increased subcortical and reduced prefrontal dopamine neurotransmission underlies psychotic symptoms and negative, affective, or cognitive dimensions respectively. However, there is limited evidence of a direct impairment within the dopaminergic system itself in affected patients, and, thus, research is focused on afferent pathways onto ventral tegmental area (VTA) dopaminergic neurons that project to different brain regions, like the prefrontal cortex and ventral subiculum, of relevance in the aforementioned conditions^1^. Psychotic symptoms are well treated by the administration of antipsychotics, which are mainly pharmacological antagonists of the dopamine D2 receptor (DRD2), but there is an urgent need to understand the molecular mechanisms underlying negative and cognitive symptoms in affected patients to develop new pharmacological approaches to treat them.

Abnormal inhibitory neurotransmission has also been reported to be another hallmark of schizophrenia and bipolar disorder. In contrast to the dopaminergic system, clear evidence of reduced mRNA and protein levels of GAD67, the enzyme that synthesizes the inhibitory neurotransmitter gamma-aminobutyric acid (GABA), has been shown in studies of postmortem patients. Interestingly, not all inhibitory interneuron subtypes are impaired, as increasing evidence points to a subgroup of interneurons, those expressing the calcium-binding protein parvalbumin (PV), to be particularly affected^2–4^. DRD2 is developmentally expressed in PV interneurons throughout the transition between adolescence and adulthood, thus gradually setting a delicate excitation-inhibition ratio, necessary for an appropriate processing of information^5–8^. It has been shown that selective DRD2 deletion from the entire PV interneuron population (PV^*DRD2KO*^) causes an increase in the excitatory tone and schizophrenia-like phenotypes in mice, including increased locomotor activity, reduced motivation, and cognitive impairment^9,10^. However, clear evidence is lacking regarding the role of DRD2 expressed by PV interneurons with particular phenotype domains observed in PV^*DRD2KO*^ animals to associate these domains to the expression of PV interneurons in circumscribed brain regions, like PFC or ventral subiculum. Therefore, a clear understanding of the specification and developmental trajectories of cortical interneuron subtypes may shed light on the etiology of psychiatric diseases.

Cortical interneurons are born from the medial and caudal ganglionic eminences (MGE and CGE, respectively) and the preoptic area (POA). The MGE is the region that mostly contributes to the cortical interneuron population, specifying either PV or somatostatin (SST) interneurons^11–13^. In spite of the fact that there are many known MGE transcription factors involved in the specification of PV and SST interneurons^14–17^, is yet unknown the precise molecular mechanisms through which these two populations are segregated. Transplant experiments have shown that the dorsal portion of the MGE gives rise primarily to SST interneurons, while ventral MGE to the PV subtype^18,19^.

Nkx6.2 is a transcription factor expressed in the ventral region of the lateral ganglionic eminence (LGE), dorsal MGE^20^, the POA and in the preoptic hypothalamic region (POH)^18^. By fate mapping and lineage tracing studies, it has been shown that Nkx6.2 primarily contributes to SST interneurons^21,22^. However, the precise contribution and subtypes of Nkx6.2-derived interneurons remain not clearly known.

To characterize in detail the population of Nkx6.2 expressing cells, we performed a lineage tracing study by a non-inducible BAC-Cre recombinase transgenic mouse line. We found that more than one-third of the total SST cortical interneurons were generated from Nkx6.2 expressing domains. Interestingly, we identified and quantified a population of cortical interneurons derived from Nkx6.2 in the late stage of neurogenesis that are committed to be of the PV subtype, with a peak contribution at embryonic day 17 (E17). Moreover, selective DRD2 deletion from Nkx6.2 expressing progenitors in mice caused cognitive and negative phenotypes but not hyperlocomotion, reminiscent of the positive symptoms of schizophrenia or bipolar disorder. Our results highlight the relevance of Nkx6.2 expressing domains in the generation of a late-born PV interneuron subpopulation and reinforce the notion of a complex developmental subpallial molecular program to generate SST and PV interneurons from restricted MGE regions. Precise combinatorial expression of transcription factors in interneuron subsets is required to avoid pathologic phenotypes and accomplish fine-tuned brain functioning.

## Results

**Figure 1:**
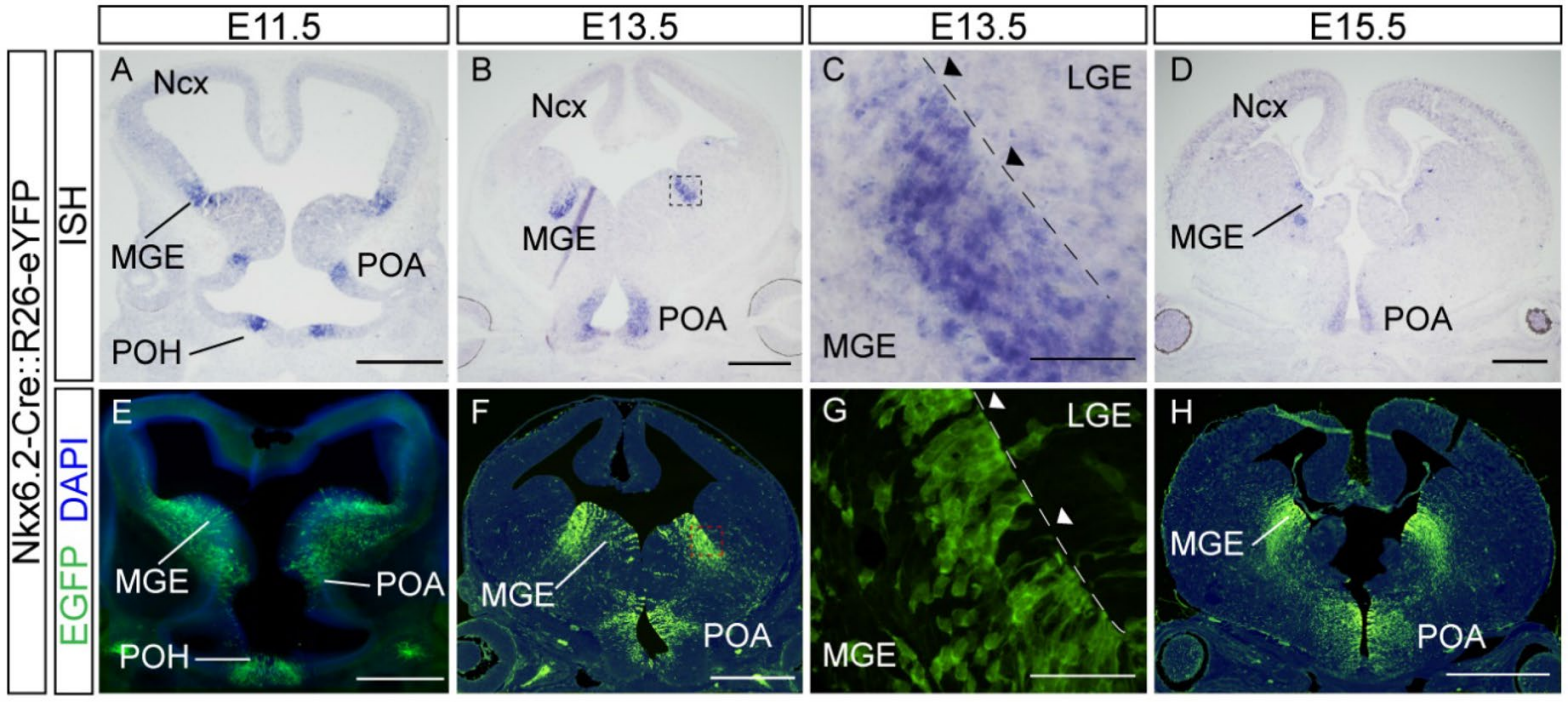
Cre recombinase activity follow the expression pattern of the endogenous Nkx6.2 mRNA. Nkx6.2 mRNA expression by in situ hybridization and immunofluorescence (IF) in Nkx6.2-Cre::R26-eYFP embryo tissue. (**A**, **B**, **D**) ISH of coronal sections at E11.5, E13.5 and E15.5 respectively, showing the mRNA expression of the transcription factor Nkx6.2. (**C**) 60× magnification of the LGE–MGE boundary from the E13.5 ISH. (**E**, **F**, **H**) IF of coronal sections at E11.5, E13.5 and E15.5 respectively, showing Nkx6.2 derived neurons. (**G**) 60× magnification of LGE-MGE boundary from the E13.5 IF. Scale bar A,B,D,E,F,H: 500 µm; C,G: 50 µm.

To characterize Nkx6.2-derived cells, we generated a transgenic line that expresses Cre recombinase under the transcriptional control of the Nkx6.2 promoter region by BAC recombineering. In order to study if the expression pattern of the Cre gene was confined to Nkx6.2 mRNA expressing domains, we analyzed the activity of Cre recombinase by mating the transgenic Cre lines with a YFP reporter^23^ and then performed immunofluorescence experiments using an EGFP specific antibody in brain sections at different time points during development. Our results showed that the expression of the Cre recombinase in the selected mouse line along rostro-caudal brain sections is restricted to telencephalic domains where Nkx6.2 mRNA is expressed (Fig. 1 and Supplementary Fig. S1). At embryonic day 11.5 (E11.5), the earliest time point analyzed, Nkx6.2 mRNA and YFP reporter expression co-localized in the ventricular zone of the prospective ventral LGE, the dorsal MGE, the POA and the POH (Fig. 1A,E). At E13.5, Nkx6.2 mRNA was expressed in the ventral LGE, the dorsal MGE, POA and POH and YFP reporter protein followed the same expression pattern (Fig. 1B,F). A close analysis revealed that Nkx6.2 mRNA was not evenly expressed throughout the ventricular zone of the dorsal MGE or ventral LGE, as progenitors of low or null ISH signal were encompassed by progenitors showing high ISH signal (Fig. 1C). In line with these results, immunofluorescence performed on adjacent brain sections to those where ISH was performed showed YFP expressing progenitors, due to Cre expression, (Fig. 1G) in a similar pattern to the one observed in the ISH (Fig. 1C). Analysis at E15.5 showed that the reporter recapitulates the endogenous expression of the Nkx6.2 mRNA and a stream of migrating interneurons was visible in the marginal and subventricular zone of the developing neocortex (Fig. 1D,H). In neonate animals (post-natal day 0, P0), neural stem cells from the ventricular zone of the lateral ventricle expressed YFP (Supplementary Fig. S1), as previously described^24^. Brain sections from postnatal day 30 (P30) animals showed YFP-labelled cells in the cerebral cortex, preoptic area, hypothalamus, hippocampus, and olfactory bulb (Supplementary Fig. S1, S2). In addition, these brain sections showed that Nkx6.2 expressing progenitors generated either oligodendrocytes or neurons, as YFP positive cells were co-labelled with the oligodendroglial marker CC1 or the neuronal marker NeuN, but not with glial fibrillary acidic protein (GFAP), an astrocytic marker (Supplementary Fig. S3).

**Figure 2:**
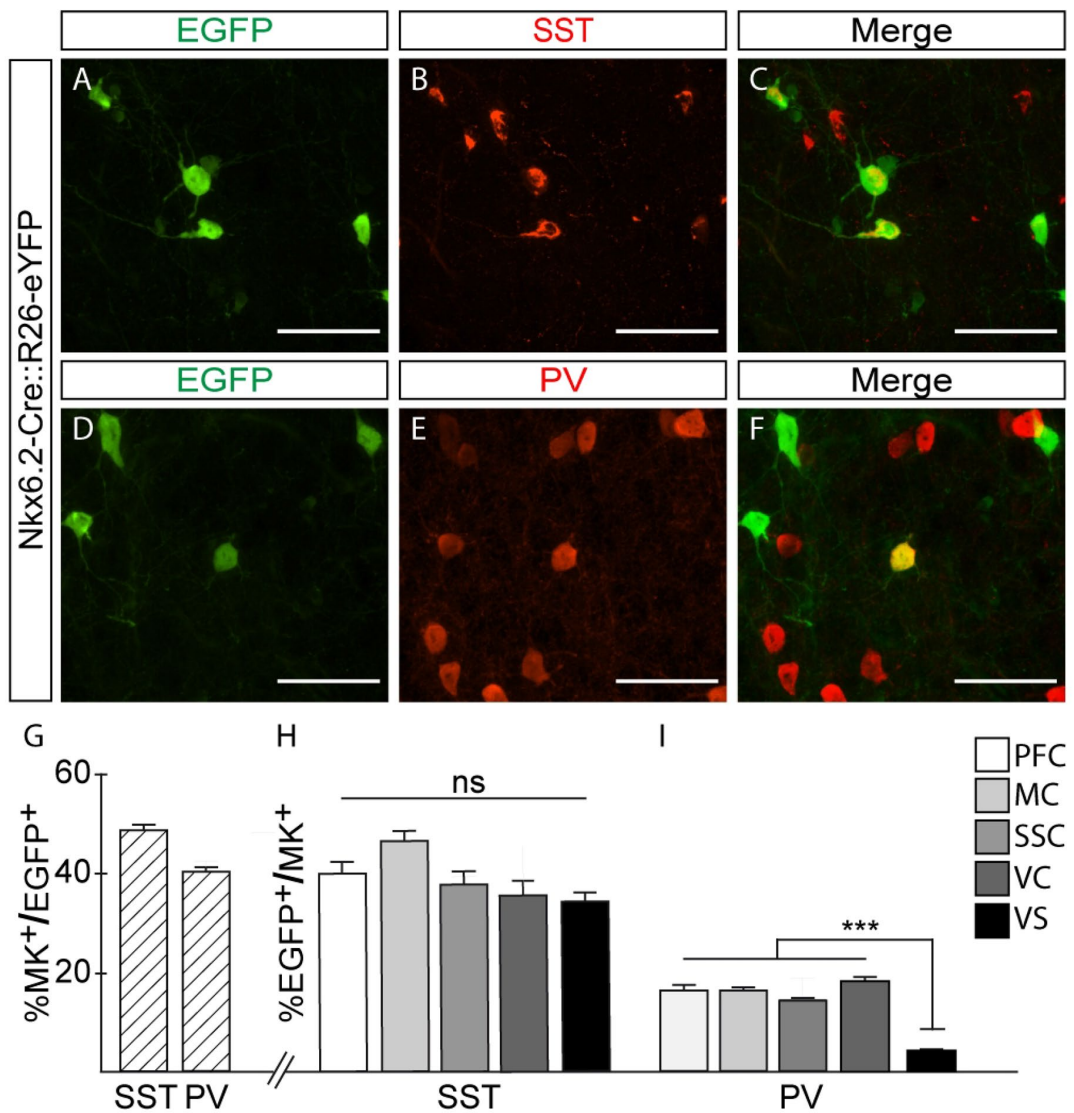
Nkx6.2 generates somatostatin and parvalbumin interneurons. (**A**–**F**) Immunofluorescence of coronal sections from Nkx6.2-Cre::R26-eYFP in P30 mice. (**A**–**C**) Immunofluorescence for (**A**) EGFP, (**B**) SST and (**C**) merged channels showing somatostatin interneurons derived from Nkx6.2 expressing progenitors. (**D**–**F**) Immunofluorescence for (**D**) EGFP, (**E**) PV and (**F**) merged channels showing parvalbumin interneurons derived from Nkx6.2 progenitors. (**G**) Quantification of marker (SST or PV) interneurons from total Nkx6.2 derived interneurons (MC, SSC n = 10–12, VC n = 3–4). (**H**,**I**) Quantification of Nkx6.2 derived interneurons from total marker (SST o PV) population (MC, SSC n = 11–12, VC, PFC, VS n = 3–4). Images: 60× magnifications from motor cortex; Bregma 0.5 mm. Scale bar: 50 µm. PFC: Pefrontal Cortex; MC: Motor cortex; SSC: Somatosensory cortex; VC: Visual cortex; VS: Ventral Subiculum. Values are presented as mean ± s.e.m. ***p < 0.001. One way ANOVA (F = 6.505; *p* = 0.0006).

It was previously shown that the dorsal MGE specifies preferentially cortical SST interneurons^18^. As our mouse line labelled a substantial number of neurons in the cortex and Nkx6.2 is expressed in the MGE with a high-dorsal to low-ventral graded expression (see Fig. 1), we determined if YFP labelled cortical neurons were of the SST subtype (Fig. 2A–C). Our immunofluorescence experiments followed by quantifications showed that 47.1 ± 1.4% of the total population of Nkx6.2-derived cortical interneurons expressed SST (SST^+^) at P30 (Fig. 2G). Remarkably, 41.1 ± 1.3% of the Nkx6.2-derived cortical interneurons were of the PV subtype (PV^+^) (Fig. 2D–F,G ). Quantification of SST expressing Nkx6.2-derived neurons from the total SST interneuron population (EGFP^+^/SST^+^) showed a comparable contribution throughout prefrontal, motor, somatosensory or visual cortices and ventral subiculum (Fig. 2H). However, quantification of PV expressing Nkx6.2-derived neurons from the total PV interneuron population (EGFP^+^/PV^+^) showed a comparable contribution throughout cortical regions but a significantly reduced contribution in the ventral subiculum compared to cortical regions. (Fig. 2I).

**Figure 3:**
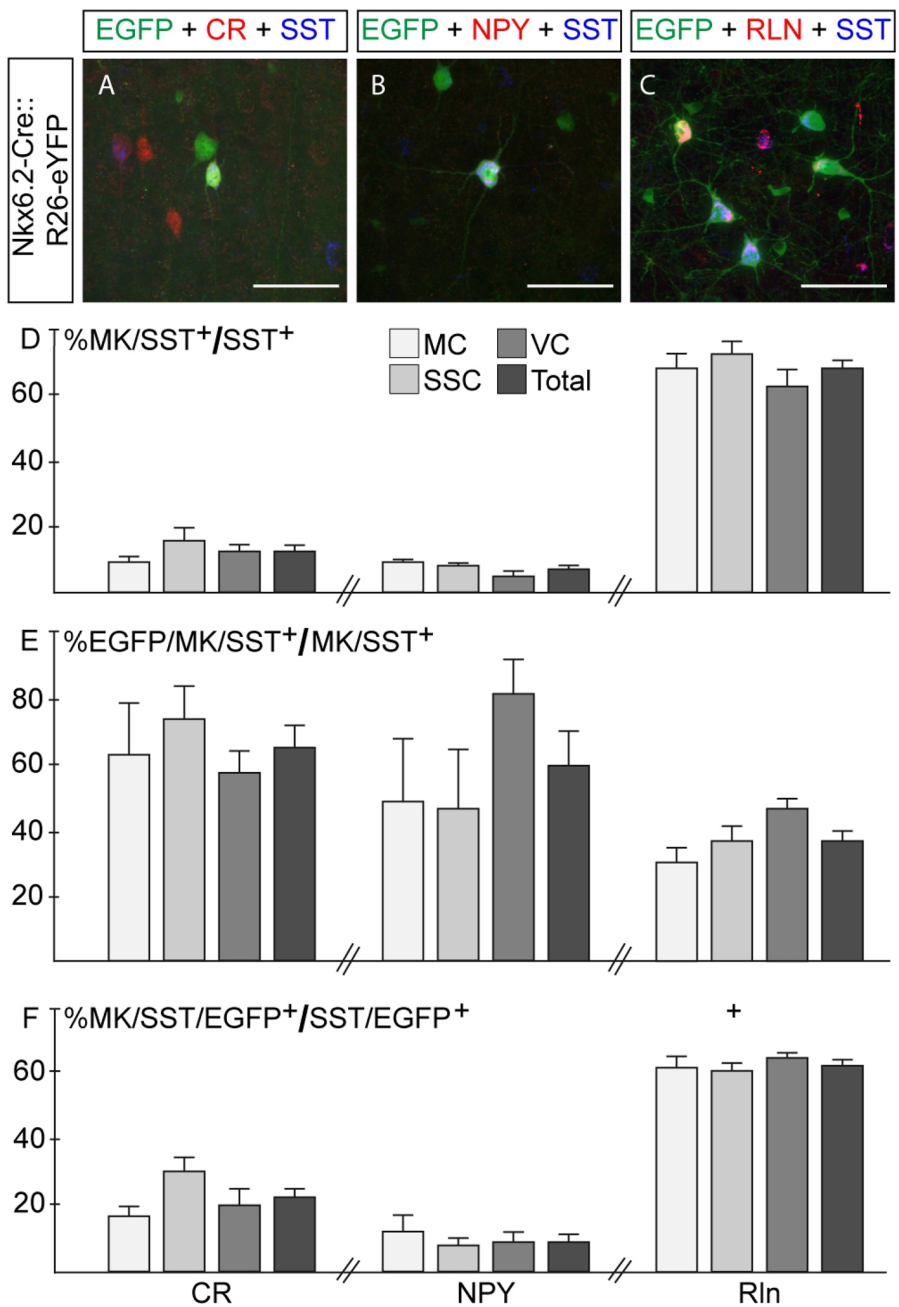
All Nkx6.2 derived somatostatin interneurons co-express calretinin, reelin or NPY. (**A**–**C**) Immunofluorescence of coronal sections from Nkx6.2-Cre::R26-eYFP in P30 mice. Immunofluorescence for EGFP (green), somatostatin (blue) and (**A**) CR, (**B**) NPY and (**C**) Rln (red). (**D**) Quantification of SST/Marker^+^ (CR, RLN or NPY) interneurons from total SST^+^ population (n = 3–4). (**E**) Quantification of Nkx6.2/SST/Marker^+^ (CR, RLN or NPY) interneurons from total SST/Marker^+^ (CR, RLN or NPY) population (n = 3–4). (**F**) Quantification of Nkx6.2/SST/Marker^+^ (CR, RLN or NPY) interneurons from total Nkx6.2/SST^+^ interneuron population (n = 3–4). Images: 60X magnification from motor cortex; Bregma 0.5 mm. Scale bar: 50 µm. Values are presented as mean ± s.e.m.

**Figure 4:**
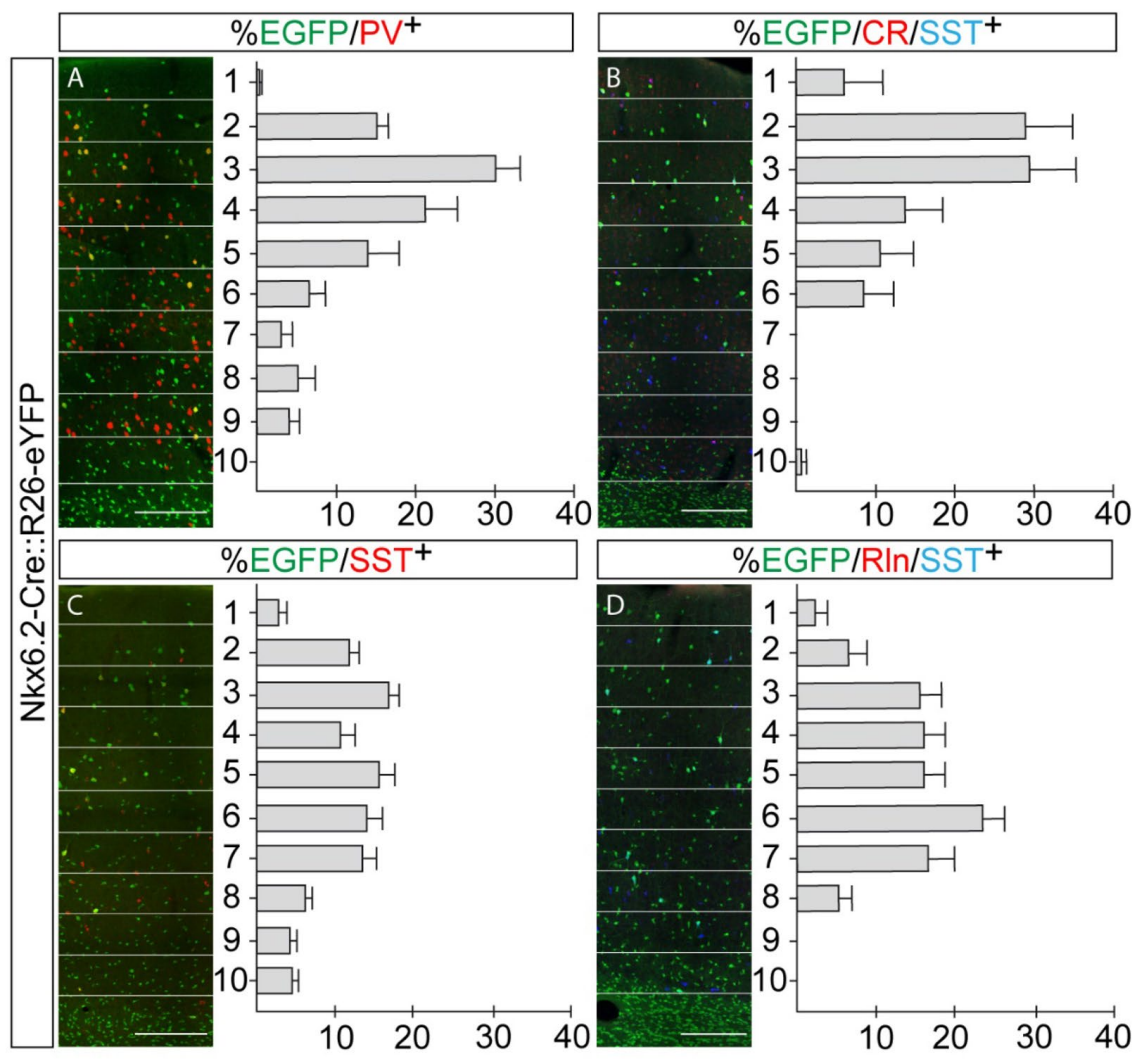
Laminar distribution of Nkx6.2 derived interneurons. (**A**) Immunofluorescence for EGFP (green) and PV (red) and the corresponding laminar distribution of PV interneurons derived from Nkx6.2 (n = 3). (**B**) Immunofluorescence for EGFP (green), CR (red) and SST (blue) and the corresponding laminar distribution of SST/CR^+^ interneurons derived from Nkx6.2 (n = 3–4). (**C**) Immunofluorescence for EGFP (green) and SST (red) and the laminar distribution of SST interneurons derived from Nkx6.2 (n = 3). (**D**) Immunofluorescence for EGFP (green), Rln (red) and SST (blue) and the corresponding laminar distribution of SST/RLN^+^ interneurons derived from Nkx6.2 (n = 3–4). Single images were taken from motor cortex at Bregma 0.5 mm with a 20X magnification and composed. The width of the cortex was divided into 10 equal bins for laminar analysis. Scale bar: 200 µm. Values are presented as mean ± s.e.m.

SST interneuron may co-express other markers (MK), like calretinin (CR), neuropeptide Y (NPY) or reelin (Rln), among others. We thus characterized in detail the SST^+^ population, regardless the YFP expression, and found that 12.8 ± 1.5% of SST^+^ interneurons expressed CR (Fig. 3A,D), 7.1 ± 0.9% expressed NPY (Fig. 3B,D) and 67.4 ± 2.5% expressed Rln (Fig. 3C,D), while the remaining SST^+^ interneurons, about 12%, were negative for any of these three markers. When we analyzed the contribution of Nkx6.2 Cre line within each marker (EGFP^+^/MK^+^/SST^+^ from the total MK^+^/SST^+^ population), we found that the Nkx6.2 domain contributed with 64.4 ± 6.6% for the total CR^+^/SST^+^ population, 59.2 ± 9.9% for NPY^+^/SST^+^ population and 36.3 ± 3.1% for Rln^+^/SST^+^ population (Fig. 3E). Finally, we found that 23.8 ± 2.9% of the EGFP^+^/SST^+^ interneurons co-expressed CR, 65.7 ± 1.4% co-expressed Rln and 10.1 ± 1.7% co-expressed NPY (Fig. 3F) and therefore, all EGFP^+^/SST^+^ interneurons co-expressed any of these three polypeptides. Layer distribution within the EGFP^+^/SST^+^ population showed that those interneurons that co-express CR are settled primarily in supragranular layers with virtually no interneurons of this subtype in deep layers (Fig. 4B) while those co-expressing reelin were mainly placed in perigranular layers (Fig. 4D).

**Figure 5:**
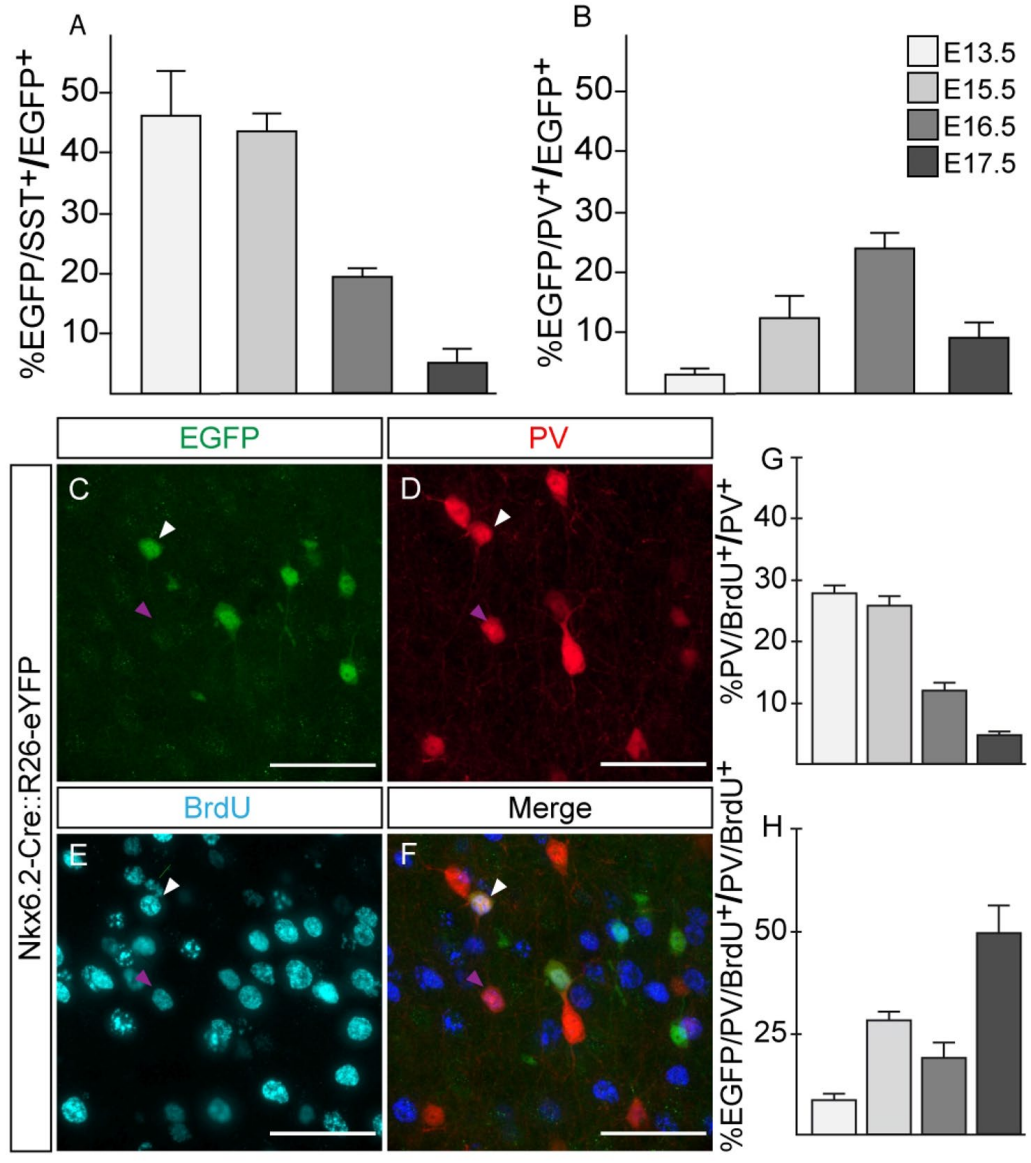
Parvalbumin and somatostatin, Nkx6.2 derived interneurons, follow an opposite timed developmental program. (**A**) Quantification of somatostatin interneurons derived from MGE Nkx6.2 expressing progenitors respect to total Nkx6.2 transplanted neurons (n = 3 donor animals for each day). (**B**) Quantification of parvalbumin interneurons derived from MGE Nkx6.2 expressing progenitors respect to total Nkx6.2 transplanted neurons (n = 3 donor animals for each day). (**C**–**H**) Nkx6.2 derived parvalbumin interneurons are born at late developmental time points. (**C**–**F**) Immunofluorescence of coronal sections from Nkx6.2-Cre::R26-eYFP BrdU injected P30 mice for (**A**) EGFP, (**B**) PV, (**C**) BrdU and (**D**) the merged channels. White arrowheads indicate Nkx6.2^+^/PV^+^/BrdU^+^ interneuron. Purple arrowheads indicate Nkx6.2 negative PV^+^/BrdU^+^ interneuron. (**G**) Quantification of PV^+^/BrdU^+^ interneurons from total PV population (n = 3–5). (**H**) Quantification of PV^+^/BrdU^+^ interneurons derived from Nkx6.2 progenitors from total PV^+^/BrdU^+^ population (n = 3–5). Images: 60× magnification from motor cortex; Bregma 0.5 mm. Scale bar: 50 µm. Values are presented as mean ± s.e.m.

It has been shown that CR^+^/SST^-^ cortical interneurons are specified from CGE progenitors^25^. Nkx6.2 is not expressed in this region and, accordingly, few EGFP^+^/SST^-^ interneurons were labelled by CR or other CGE or POA- derived cortical interneuron markers like VIP, Rln or NPY and none of them were EGFP^+^/SST^-^/NPY^+^/Rln^+^ (Supplementary Fig. S4). These results suggest that neither the POA nor the CGE contributed with a substantial number of cortical interneurons from this transcription factor, and, therefore, all Nkx6.2-derived cortical interneurons seem to be generated from the MGE. To evaluate this hypothesis with a different experimental strategy, we transplanted MGE or POA dissociated tissue from E13.5, E15.5, E16.5 or E17.5 Nkx6.2 YFP embryos into a P0 (neonatal) cortex and analyzed Nkx6.2-derived interneurons at P30. Our results showed that Nkx6.2-derived progenitors gave rise to cortical PV or SST interneurons. Nkx6.2-derived SST interneurons exhibited a peak contribution from E13.5 MGE tissue with a sustained decline afterward (Fig. 5A). In contrast, Nkx6.2-derived PV interneurons showed a moderate contribution from E13.5 MGE tissue with a marked increase afterwards, reaching a peak at E16.5 (Fig. 5B). A cortical layer distribution analysis showed that Nkx6.2-derived SST interneurons are homogenously distributed along all cortical layers (Fig. 4C). However, EGFP^+^/PV^+^ interneurons showed a preference for supragranular layers (Fig. 4A). Interestingly, we were not able to find interneurons derived from POA transplants, suggesting that most POA Nkx6.2 progenitors did not migrate into the cortex.

As we found that the Nkx6.2 domain generated a considerable proportion of PV interneurons with a peak at E16.5, we next study the timing at which EGFP^+^/PV^+^ interneurons were generated during development by birthdating experiments with BrdU (Fig. 5C–F). In the time frame analyzed, BrdU labelled PV interneuron population (BrdU^+^/PV^+^) showed a peak of contribution at E13.5 and then the proportion decayed towards late gestational time points (Fig. 5G). However, when EGFP^+^/BrdU^+^/PV^+^ interneurons were contrasted with the total BrdU^+^/PV^+^ population, the proportion of PV interneurons derived from Nkx6.2- expressing progenitors showed an opposite contribution trend, with a peak at E17.5 (Fig. 5H). Therefore, these results, in line with previous transplant experiments, showed that dorsal MGE Nkx6.2-derived PV interneurons followed a specific developmental time sequence and were preferentially generated at later neurodevelopmental time.

**Figure 6:**
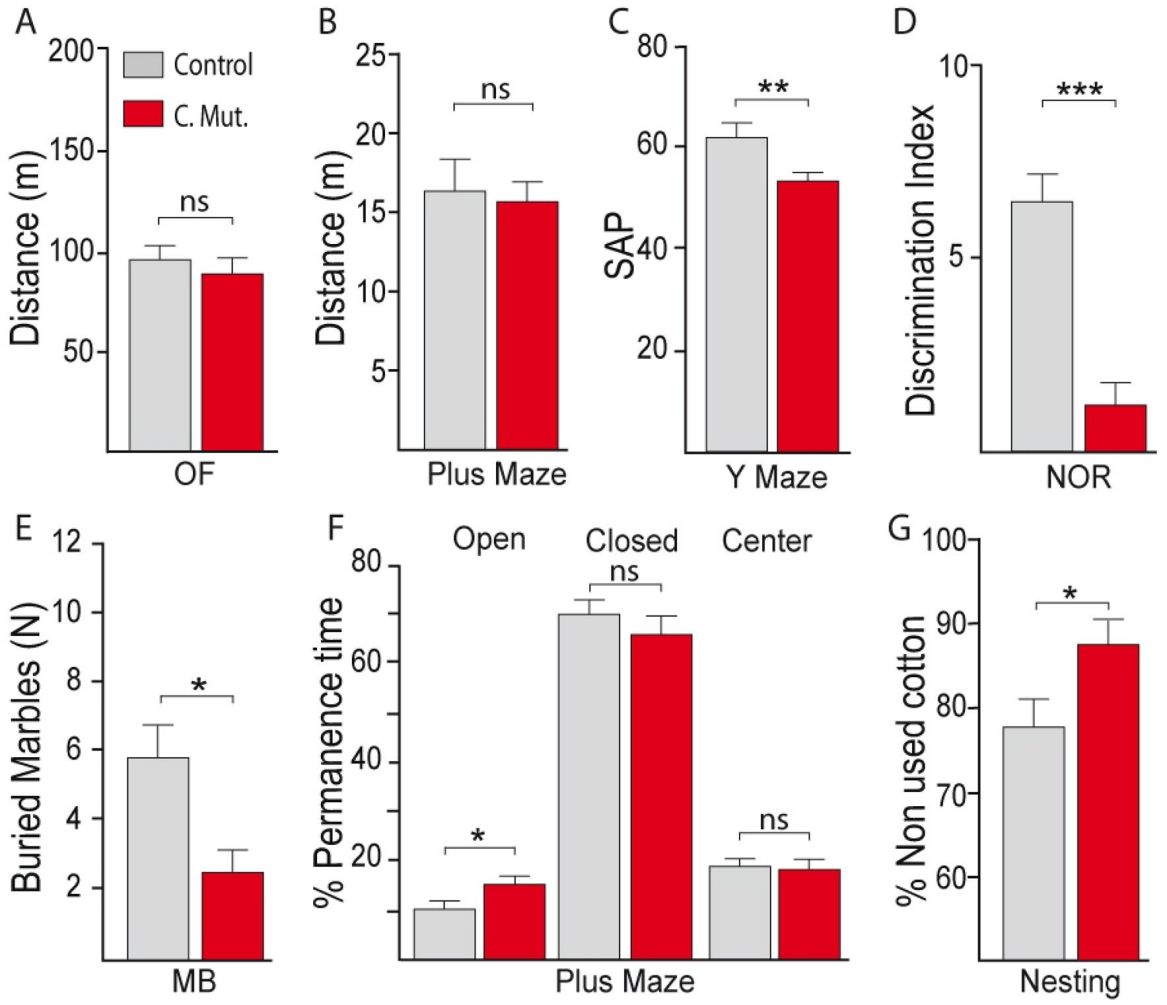
Behavioral phenotypes in DRD2 conditional mutants. (**A**, **B**) Locomotor activity in the open field (OF) and plus maze of adult (P60) control (grey) and mutant animals (red). (**C**) Spontaneous alternation performance (SAP) in the Y maze. (**D**) Discrimination index in the novel object recognition (NOR) test. (**E**) Number of buried marbles in the marble burying (MB) test. (**F**) Percentage of permanence time in the open, closed, or center areas in the plus maze (PM) test. (**G**) % of non used cotton in the nesting test. Values are presented as mean ± s.e.m. *p ≤ 0.05, **p ≤ 0.01, ***p < 0.001, ns, not significant. n = 10–20 animals per group. Unpaired t test (**A**–**D**, **F**); Mann–Whitney (**E**, **G**).

Impaired late-born PV interneurons are thought to mediate pathological behavioral phenotypes in an animal model of schizophrenia^26^. Given that we observed Nkx6.2-generated PV interneurons in late gestational time points and that deletion of DRD2 from PV interneurons causes schizophrenia-like phenotypes^9^, we wondered whether Nkx6.2-derived interneurons lacking DRD2 may result in abnormal behavioral phenotypes. An open field experiment and locomotor activity in the plus maze test showed that there were no significant differences between P60 control (DRD2^flox/flox^) and conditional mutants (Nkx6.2 Cre-DRD2^flox/flox^) in total distance travelled (Fig. 6A,B and Supplementary Table S1). However, conditional mutants showed abnormal anxiety levels, as they buried significantly less marbles in the marble-burying test and spent significantly more time in the open arms of a plus maze (Fig. 6E,F and Supplementary Table S1). They also exhibited memory deficits showing significantly reduced spontaneous alternation in the Y maze as well as a reduced discrimination index in the novel object recognition test (Fig. 6C,D and Supplementary Table S1). Finally, a motivation deficit was observed in mutants, as they used significantly less cotton compared to control animals to build a nest (Fig. 6G and Supplementary Table S1).

## Discussion

With the aim of characterizing Nkx6.2 expressing progenitors avoiding constraints of inducible systems, we followed a non-inducible approach by the generation of a transgenic Cre line by BAC recombineering, allowing us to characterize a vast population of interneuron subtypes derived from this transcription factor. Different genetic strategies were followed to identify interneuron subtypes, morphology, and intrinsic properties of Nkx6.2-derived expressing progenitors^21,22,24^. Although a compound transgenic strategy was used previously to study the contribution of cortical interneurons from the subpallial region^21^, a detailed analysis of the different subtypes of interneurons derived exclusively from Nkx6.2 was lacking. Moreover, the number of Nkx6.2 labelled neurons using inducible genetic strategies seemed to be rather limited^22^, precluding a thorough analysis. Accordingly, a report showed that the use of tamoxifen in pregnant mice blocks neural progenitor cell proliferation which ultimately results in impaired cortical neurogenesis^27^.

Our results showed that the activity of Cre recombinase followed the endogenous Nkx6.2 mRNA expression pattern throughout development. The in-situ hybridization (ISH) study revealed that endogenous Nkx6.2 mRNA expression is not homogenous along the ventricle, showing a mosaic expression pattern, alternating high Nkx6.2 expressing progenitors with seemingly non-expressing ones. YFP expression in the Cre line followed this endogenous expression pattern. A stream of YFP-labelled migrating neurons was seen at E13.5 and E15.5 from the MGE. Of note, as our genetic strategy involves permanent labelling, migrating neurons span a wider region in the mantle zone of the MGE compared to the endogenous mRNA signal due to the permanent expression of the YFP reporter protein after recombination. At P0, the ventricular/subventricular zone of the lateral ventricles was populated with YFP-labelled cells that may contribute to the rostral migratory stream, as the olfactory bulb was populated with labelled cells, suggesting that the Nkx6.2 transgenic line recapitulates the specification of neurons in this region, as previously reported^24^. Subsequently, at P30, YFP-labelled cells were observed in different brain regions, including the cortex, POA, hypothalamus and hippocampus. Therefore, the generated Nkx6.2-Cre line mimics the endogenous expression of Nkx6.2 mRNA and labels a wide population of cells along development, populating specific brain regions.

Analysis of brain sections at P30 allowed us to identify two types of cells derived from Nkx6.2 progenitors in the cortex, including neurons and glial cells, by the expression of the neuronal marker NeuN or the glial marker CC1. Nkx6.2-derived neurons co-expressed the neuronal marker NeuN and one of the MGE-specific interneuron markers, SST or PV. We observed that all EGFP^+^/SST^+^ interneurons co-expressed either Rln, NPY or CR, but the contribution of Nkx6.2 Cre line to each of these subpopulations was different. The transgenic line specified around two-thirds of the SST^+^/CR^+^ and SST^+^/NPY^+^ populations and one-third of the SST^+^/Rln^+^ population. The high dorsal MGE Nkx6.2 expression may primarily drive the specification of SST^+^/CR^+^ and SST^+^/NPY^+^ interneurons, while SST^+^/Rln^+^ interneurons may be generated by middle to ventral MGE Nkx6.2 expressing progenitors, coincident with a reduction in the expression of Nkx6.2 mRNA and transgene expression. However, further research will be needed to clearly establish the role of Nkx6.2 in the specification of different subtypes of SST interneurons.

Nkx6.2-derived SST interneurons were evenly distributed along all cortical layers, suggesting that this population of interneurons may be born from early to late developmental time points. However, within the SST^+^ subpopulation, those expressing CR showed a clear trend toward supragranular layers while those expressing Rln settled preferentially in perigranular layers, suggesting that the Nkx6.2-derived SST^+^/CR^+^ population may be born after the Nkx6.2-derived SST^+^/Rln^+^ population. EGFP^+^/PV^+^ subpopulation occupied preferentially supragranular layers, suggesting that they were born during late stages of neurogenesis.

Transplant experiments confirmed that Nkx6.2 progenitors gave rise to both SST and PV interneurons and showed that the generation of these two types of neurons followed opposite temporal sequences. Nkx6.2-derived somatostatin interneurons were born primarily at early developmental time points, compared to Nkx6.2-derived parvalbumin interneurons, which were mainly generated at late developmental stages.

The POA is a source of cortical interneurons^28,29^. In addition to the LGE/MGE boundary, Nkx6.2 is also expressed in the POA and POH. However, our results suggest that Nkx6.2 progenitors from the POA do not migrate into the cortex but settle in the preoptic area and hypothalamus in P30 animals, as we were not able to find neurons derived from the POA in the cortex from transplant experiments.

By birthdating, transplant and layer distribution quantification experiments, we determined that Nkx6.2 derived parvalbumin population was preferentially generated at late developmental stages from dorsal MGE progenitors, with a peak at embryonic day 17. Therefore, Nkx6.2 expressing progenitors has the potential to give rise to both somatostatin and parvalbumin interneurons in a specific temporal sequence, as dorsal MGE progenitors preferentially generate somatostatin neurons in early developmental stages but parvalbumin interneurons during late development. Our results are in line with a report showing that a Somatostatin-Cre line gives rise to parvalbumin interneurons^30^. In this work, the authors attributed off-target recombination for this phenomenon, however, our results suggest that the dorsal MGE may have a common pool of progenitors for the temporal generation of somatostatin and parvalbumin interneurons with a complex transcriptional regulation.

Although our knowledge on cortical interneuron specification has increased in the last years, we still lack a detailed molecular program on how parvalbumin and different subtypes of somatostatin interneurons are specified from the MGE. The graded Sonic hedgehog (Shh) concentration in the ventricle may be involved in this specification. Nkx6.2 and other factors including *Ptch1*, *Hhip1* and *Gli1* have been postulated as mediators of Shh signaling. The enrichment of these factors in the dorsal region of the MGE supports this notion^31^. Accordingly, Nkx6.2 and Gli1 high dorsal MGE expression is lost in *Nestin*-Cre;Shh^f/f^ mutants^32^. Additionally, experiments in a *Six3*-Cre;*Smo*^f/f^ mice line in which the SHH signaling effector Smoothened was deleted within a subpopulation of MGE progenitors, showed a mosaic ectopic expression of Nkx6.2 in the medial and ventral region of the MGE with a reduced cortical parvalbumin population and an expansion of the cortical somatostatin population^33^. Somatostatin and parvalbumin interneurons can be generated from the same radial glia progenitor^34–36^ and Notch signaling is involved in their specification^37^. Nkx6.2 transcriptional regulation may be modulated by mutually excluding factors within the MGE. Etv1 has been proposed to play this role, as it shows high ventral to low dorsal expression^38,39^. On the other hand, Ptf1a, a transcription factor that mediates GABAergic fates in the cerebellum^40^ is expressed in ventral regions of the forebrain during development^41^ and may repress MGE Nkx6.2 activity^42^. It has been shown that the transcription factor Onecut modulates the expression of Nkx6.2 in the spinal cord^43^. Further research will be needed to precisely determine if any of these factors are involved in the transcriptional regulation of Nkx6.2, its high dorsal to low ventral MGE expression pattern and the relevance of this graded expression in the contribution of different subtypes of somatostatin and parvalbumin interneurons.

It has been reported that the administration of the methylating agent methylazoxymethanol acetate (MAM) to pregnant dams in late gestational periods causes phenotypes highly reminiscent of schizophrenia symptoms in the offspring when they reach adulthood^44^. In the MAM schizophrenia model, late-born parvalbumin interneurons populating the PFC or the ventral subiculum are particularly affected^26^. In line with these findings, we previously showed that the specific deletion of DRD2 from total parvalbumin interneuron population caused schizophrenia-like phenotypes in mice^9,10^. Using this model, however, it’s not possible to associate specific behavioral phenotypes to defined brain regions where dopamine D2 receptor has been selectively deleted, as the receptor is ablated from the entire parvalbumin population. Given that we observed a late-born PV interneuron population derived from Nkx6.2 populating preferentially the PFC and not the ventral subiculum, we asked whether the deletion of DRD2 from this subpopulation of neurons might reproduce domain-specific phenotypes within schizophrenia pathophysiology. DRD2 selective deletion from Nkx6.2 derived cells caused behavioral phenotypes within cognitive and negative domains, like impaired memory and motivation, but not those associated to positive domains, like increased locomotor activity. Cognitive and emotional deficits displayed by the Nkx6.2Cre-DRD2^flox/flox^ line may be attributed to impaired dopaminergic signaling in late-born parvalbumin interneuron populating the PFC. Impairment of the ventral subiculum parvalbumin interneuron population underlies dopamine dysregulation in schizophrenia animal models^26,45^. The low contribution of Nkx6.2 derived PV interneurons to the ventral subiculum may not be sufficient to impair the excitatory-inhibitory balance in this region, associated to a hyperdopaminergic state and to an increased locomotor activity^9^. DRD2 specific deletion in Nkx6.2Cre-DRD2^flox/flox^ line is not restricted to PV interneurons, as we showed a wide SST population derived from this transcription factor, but the contribution of Nkx6.2-derived somatostatin interneurons across analyzed brain regions were not different. However, significantly less Nkx6.2-derived parvalbumin interneurons populated the ventral subiculum compared to other cortical regions. Further research will be needed to delineate behavioral responses when PV interneuron inhibitory activity mediated by DRD2 signaling in specific brain nuclei is impaired. However, our work reinforces the notion of a confined prefrontal hypodopaminergic neurotransmission that may promote both cognitive and emotional deficits.

Abnormal inhibitory neuron development and function underlie different psychiatric diseases^46,47^. A clear understanding of MGE molecular hierarchies to specify somatostatin or parvalbumin interneurons is of utmost relevance in our way to understand the etiology of these diseases. Here we showed that the MGE domain that expresses Nkx6.2 generates both somatostatin and parvalbumin interneurons following a time and space-specific sequence and the deletion of DRD2 from Nkx6.2-derived progenitors caused domain-specific phenotypes associated to pathophysiology of psychiatric diseases.

## Methods

### Transgenic mice

The Nkx6.2 transgenic mice line was generated as described previously^21^. Briefly, mice expressing the codon-improved Cre recombinase (iCre) were generated using bacterial artificial chromosome (BAC) technology. The iCre gene was fused to the initiation codon of the Nkx6.2 gene using a PCR-based approach. BAC modification was performed in a bacterial system. Transgenic Nkx6.2-iCre mice were crossed with a YFP reporter line^23^. Cells with active Cre activity promote the excision of the stop cassette and therefore the YFP protein is expressed. Male Nkx6.2-Cre animals were used as breeders. Animals were maintained in a controlled environment (20–22 °C, 12 h light/dark cycle) and were group housed in individual ventilated cages with food and water ad libitum. For transplant experiments, Nkx6.2-Cre animals were crossed to a TdT reporter line (ai14)^48^. The day of vaginal plug was considered embryonic day 0.5 (E0.5). Experiments were performed in accordance with the Guide for Care and Use of Laboratory Animals published by the National Institutes of Health. The study was approved by the Institutional Animal Care and Use Committee of the Instituto de Biología y Medicina Experimental (IBYME, N019/2019). This study is reported in accordance with ARRIVE guidelines.

### Tissue collection

For embryo tissue collection, timed pregnant mothers were sacrificed by cervical dislocation and the E11.5, E13.5, E15.5 and E17.5 embryos were dissected in cold phosphate-buffered-saline (PBS). Brains were dissected and then fixed in 4% PFA solution overnight at 4 °C. Brains were then embedded in low melting point agarose (Invitrogen) and sectioned at 60-μm-thick coronal slices using a vibratome (Leica VT1000S). For P30 animals, mice were transcardially perfused with 4% paraformaldehyde (PFA) solution in PBS and the brain was removed and postfixed in the same fixative solution for 180 min at 4 °C. The tissue was cryoprotected sequentially in 10%, 20% and 30% sucrose solution in phosphate buffer saline (PBS) and then cut serially in a cryostat in 40 μm thick coronal brain sections.

### Immunofluorescence

Immunofluorescence was performed as previously described^9^. Bromodeoxyuridine was intraperitoneally injected into pregnant mice at a dose of 50 mg/kg of body weight in a single dose. The following antibodies were used in different combinations: rabbit anti-GFP 1/1000 (Invitrogen-Molecular Probes) and chicken anti-GFP 1/2000 (Aves Labs) to detect YFP reporter protein, rabbit anti-Parvalbumin 1/5000 (Swant), rat anti-Somatostatin 1/250 (Millipore), rabbit anti-Calretinin 1/2000 (Swant), mouse anti-Reelin 1/250 (MBL), rabbit anti-Vasoactive Intestinal Peptide 1/500 (ImmunoStar), rabbit anti-neuropeptide Y 1/1500 (Immunostar), rat anti-BrdU 1/250 (Abcam). Secondary antibodies were anti-rabbit AF488 1/400 (Invitrogen, Molecular Probes), anti-chicken AF488 1/400 (Invitrogen, Molecular Probes), anti-mouse AF488 1/400 (Jackson ImmunoResearch) (anti-rabbit AF555 1/400 (Invitrogen, Molecular Probes), anti-rat AF594 1/400 (Jackson ImmunoResearch), anti-rat AF647 1/400 (Jackson ImmunoResearch). Images were acquired using an Olympus IX83 microscope.

### In situ* hybridization*

In Situ Hybridization was performed as previously described^9^. The Nkx6.2 cDNA probe was generated using the following primers: 5′-GCTAAAAAGAAGCAAGACTCGG-3′ and 5′-CTCCGTATGGAAGAAACACGAT-3′.

### Cell transplantation

The afternoon before the transplantation, pregnant dams were intraperitoneally injected with a single dose of 50 mg/kg of BrdU. The following day, pregnant dams were sacrificed, embryos were dissected in cold Krebs [126 mM NaCl (Anedra), 2,5 mM KCl (Cicarelli), 1,2 mM NaH2PO4 (Anedra), 1,2 mM MgCl2 (Cicarelli), 2,1 mM CaCl2 (Cicarelli), 1,1 mM Glucose (Gibco), 1,44 mM Sodium Bicarbonate (Biopack)] and double transgenic embryos were embedded in low melting point agarose (Invitrogen). Brains were sectioned at 300-μm-thick coronal slices using a vibratome (Vibratome) and sections including the MGE or POA were selected to dissect out the dorsal MGE (dMGE) or POA. The dissected tissue from each embryo was placed in a separated eppendorf containing cold Leibovitz’s L-15 Medium (Gibco). The tissue was then mechanically dissociated into a single cell suspension by repeated pipetting. The dissociated cells were precipitated by centrifugation (3 min at 0,5 g), resuspended in 5 µl of L-15 + Dnase I grade II 100 µg/ml (Sigma) and kept on ice until use. 1,2 µl of the concentrated cell suspension was loaded at 0,5 µl/min into a G30 needle that was connected to a 5 µl Hamilton. P0-P3 host mice were anesthetized by hypothermia and placed on aluminum foil cooled with ice and under a magnifying glass for surgery. The pipettes were set in a stereotactic injection apparatus which was also connected to an injection pump in order to control the flow of the cell suspension. Injections were made in neonates in a region that spans the motor and somatosensory cortex, slightly lateral to the sagittal suture and 1 µl of the cell suspension was injected at 0.2 µl/min. After the surgery, animals were recovered on a heating pad and then put back with a foster mother. After 30 days of surgery, animals were perfused, and brain tissue analyzed as described in previous sections.

### Quantification

The extent of colocalization between the reporter and the interneuron specific marker/markers were determined as follow. 40-μm-thick coronal brain slices of Nkx6.2-Cre::R26-eYFP P30 animals were immunostained for GFP and interneuron specific markers. The prefrontal (Bregma 1.78 mm), motor (Bregma 0.62 mm), somatosensory (Bregma − 1.70 mm) and visual (Bregma − 2.46 mm) cortex and ventral subiculum (Bregma − 3.40) were photographed (Olympus IX83) in single images at a specific magnification and then assembled. Images spanning all cortical areas were used to perform quantifications. Images were processed using ImageJ and counted in Adobe Photoshop. Data are presented as mean ± SEM.

### Behavioral experiments

Behavioral experiments were conducted during the dark phase of the light/dark cycle between 6 and 11 pm and performed by an observer blind to genotype. DRD2^flox/flox^ male animals were used as the control group. Nkx6.2Cre-DRD2^flox/flox^ male animals were used as the conditional mutant study group. All tests were videotaped using a computer-assisted video acquisition software (OBS Studio, Open Broadcaster Software). The order of tests was as follows: open field (locomotor activity), elevated plus maze (anxiety), marble burying test (anxiety), Y-maze (spatial memory), novel object recognition test (NOR, memory), and nesting test (motivation). Briefly, each behavioral test was performed as follow:

#### Locomotor activity

Spontaneous locomotor activity in an open field was analyzed using ANY-maze Video Tracking System (Stoelting). Total distance travelled (horizontal activity) was analyzed.

#### Elevated plus maze

The elevated plus maze was used to study anxiety related behavior. The apparatus consists of a cross-shaped platform (four arms faced two to two with 30 cm length and 8 cm width each arm, elevated 50 cm from the floor. Two opposing arms are protected by walls (without roof) and the other two are left unprotected. The open arms represent an aversive environment for the mouse. The proportion of time spent in open arms during 8 min. is considered a measure of anxiety. Total distance travelled in the plus maze was also analyzed as a measure of the locomotor activity.

#### Marble burying

Empty home cages (30 × 24 × 15 cm) were filled with woodchip bedding up to 5 cm from the cage floor, and twelve marbles were positioned in a grid pattern throughout the cage. Mice were allowed to freely explore the cage for 30 min. Afterward, the number of successfully buried marbles was counted. A marble was defined as buried when < 30% of its surface was visible. After the test time period, a photograph was taken to analyze the result.

#### Spontaneous alternation in Y maze

Testing was carried out on a Y shaped maze (35 cm length and 5 cm width each arm). Mice were placed into the end of one arm and allowed to freely explore for 7 min. The sequence of arm entries was recorded. The spontaneous alternation behavior was calculated as the number of triads containing entries into all three arms divided by the total number of entries.

#### Novel object recognition test

A mouse was placed in an empty arena and was allowed to explore it for 5 min. 24 h later, during familiarization session, the mouse was presented with two identical objects and was allowed to freely explore them for 5 min. Three hours later, during the test session, one of the two objects was replaced by a new one and mice were allowed to explore them for 3 min. The amount of time taken to explore the new object provides an index of recognition memory. Total locomotion was also determined during the test.

#### Nesting test

To study the motivation to build a nest, one piece of pressed cotton (approximately 3 g each), was introduced in a cage in which a mouse was individually housed. Each piece of pressed cotton was weighed before the test. After the test, the unthreaded and identifiable pieces of nesting material were weighted. Pictures of the nests were also taken at the end of the experiment.

### Experimental design and statistical analysis

In this work, no human participants were involved on the study. A minimum of 3 individuals were used to perform each experiment. The contribution of Nkx6.2 to the somatostatin and parvalbumin cortical population was analyzed with 10–12 P30 animals, except for the visual cortex, where 4 individuals were used. For developmental experiments, at least three embryos of each embryonic developmental time were used (E11.5, E13.5, E15.5 and 17.5). Statistical analysis was carried out in GraphPad Prism software. The data is presented as mean ± s.e.m. Data were evaluated by t test or one way ANOVA, followed by post-hoc tests. A *p* < 0.05 was considered significant. Mann Whitney test (*p* < 0.05) was used to analyze nesting and marble burying test.

### Supplementary Information

Supplementary Information.

## Data Availability

Quantification of interneuron population data is provided throughout the work. Detailed results of each behavioral test is provided in a table as Supplementary information.
