# Supplementary material for: Selective dopamine D2 receptor deletion from Nkx6.2 expressing cells causes impaired cognitive, motivation and anxiety phenotypes in mice
Source: Sci Rep. 2023 Nov 9;13:19473. doi: 10.1038/s41598-023-46954-8 (PMC10636105; doi:10.1038/s41598-023-46954-8)
Supplement: Supplementary file 1 — Supplementary Information. [file 41598_2023_46954_MOESM1_ESM.pdf]

Selective dopamine D2 receptor deletion from Nkx6.2 expressing cells causes impaired cognitive, motivation and anxiety phenotypes in mice.

Lucila Bechelli, Eugenia Tomasella, Sofia Lopez Cardoso, Martina Belmonte and Diego Gelman.

Supplementary information.

Supplementary Figure S1. Immunofluorescences in coronal section from Nkx6.2-Cre::R26-eYFP mice showing Nkx6.2 derived neurons. (A) Coronal section at Bregma 0.5 mm showing ventricular-subventricular zone (VGZ) derived Nkx6.2 neurons amongst others. Image: 10X magnification. Scale bar: 1 mm. (B, C) Coronal sections from olfactory bulbs at (B) Bregma 4.28 mm and (C) Bregma 3.92 mm. Images: 4X magnification. Scale bar: 200 µm. Gl: Glomerular layer of the olfactory bulb. GrO: Granular cell layer of the olfactory bulb.

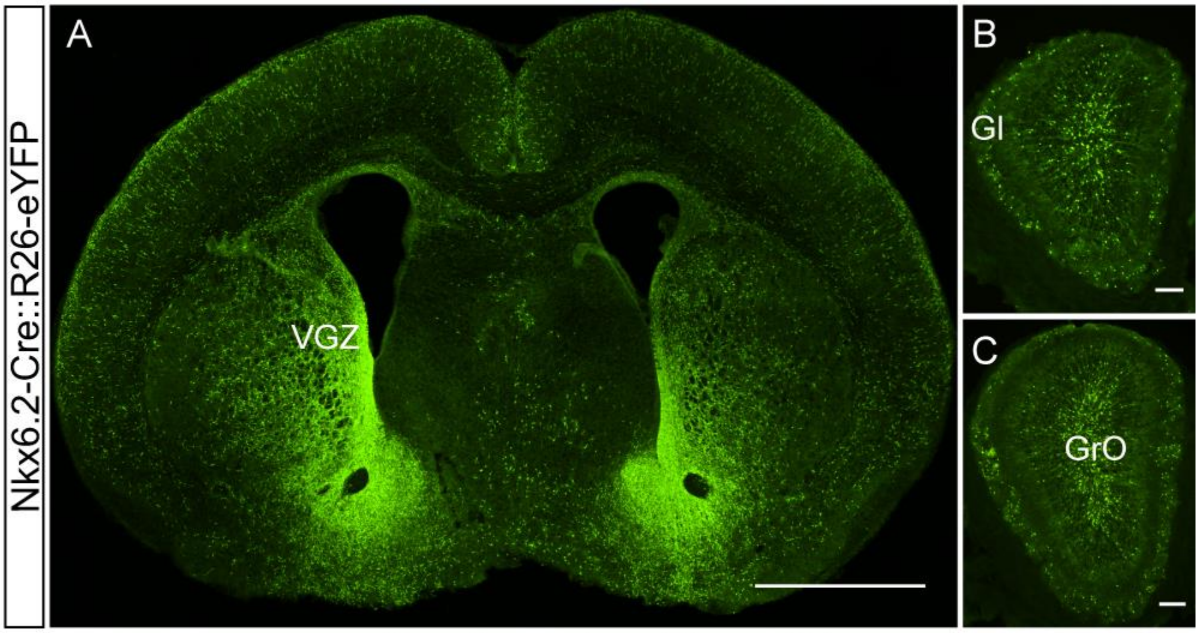

Supplementary Figure S1

Supplementary Figure S2. Nkx6.2 derived cells populates different brain regions. (A-D) Distribution of Nkx6.2 derived neurons in the brain of Nkx6.2-Cre::R26-eYFP P30 mice. (A) Motor and somatosensory cortex, Bregma 0.50 mm. (B) Preoptic area, Bregma -0.34 mm. (C) Hippocampus, Bregma -1.46 mm. (D) Hypothalamus, Bregma -1.70 mm. Images: 10X magnification and composed in a single one. Scale bar: 200 µm.

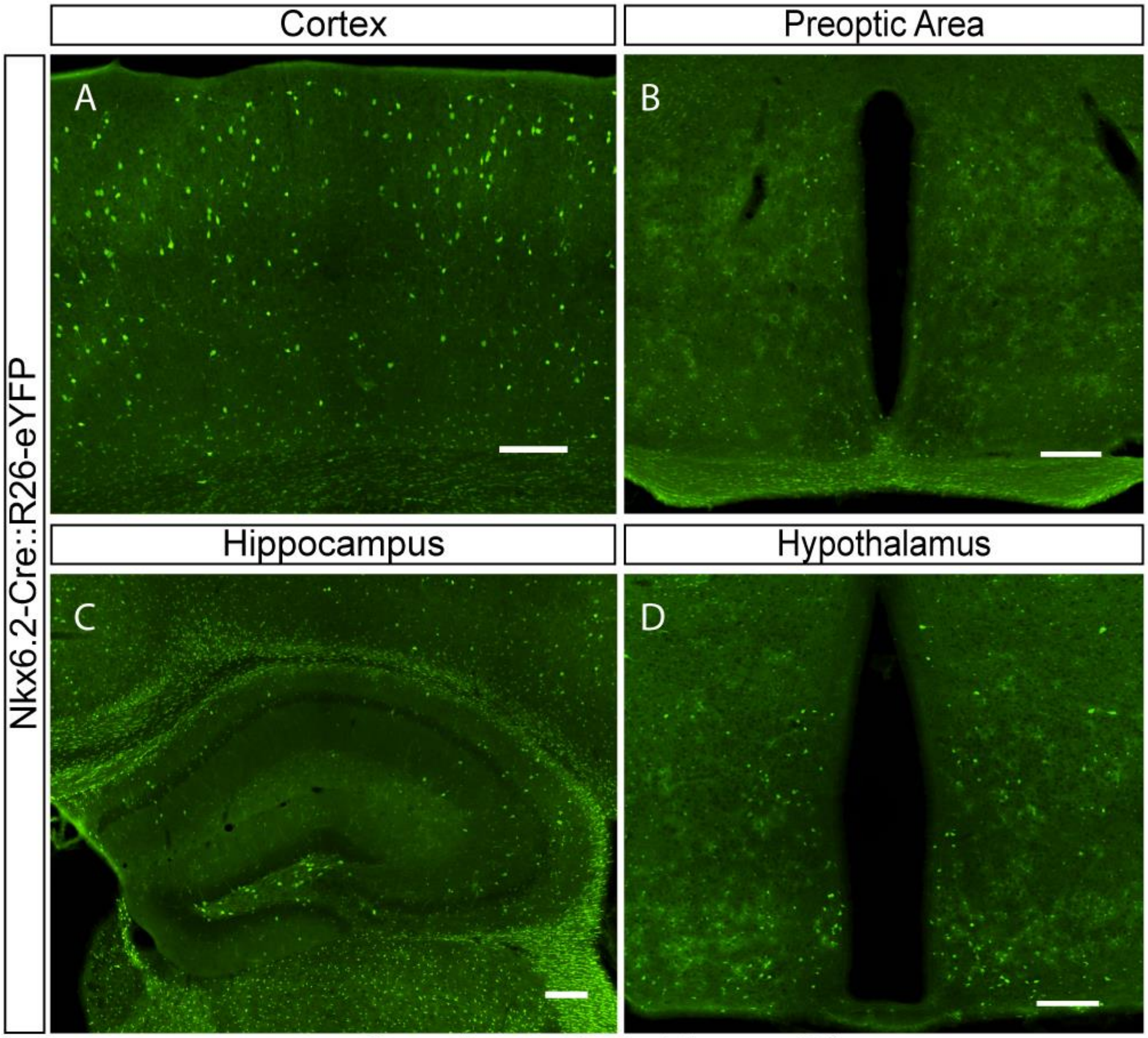

Supplementary Figure S2

Supplementary Figure S3. Nkx6.2 generates both glial cells and neurons. (A-D) Immunofluorescence for (A) EGFP, (B) NeuN, (C) GFAP and (D) merged channels in the corpus callosum (n=3). (E-H) Immunofluorescence for (E) EGFP, (F) NeuN, (G) CC1 and (H) merged channels in the corpus callosum (n=3). White arrowheads indicate oligodendrocytes derived from Nkx6.2 progenitors. (I-L) Immunofluorescence for (I) EGFP, (J) NeuN, (K) CC1 and (L) merged channels in layers II-III from motor cortex (n=3). White arrowheads indicate Nkx6.2 derived neurons whereas grays indicate Nkx6.2 derived oligodendrocytes. Images: 60X magnification; Bregma 0.5 mm. Scale bar: 50 µm.

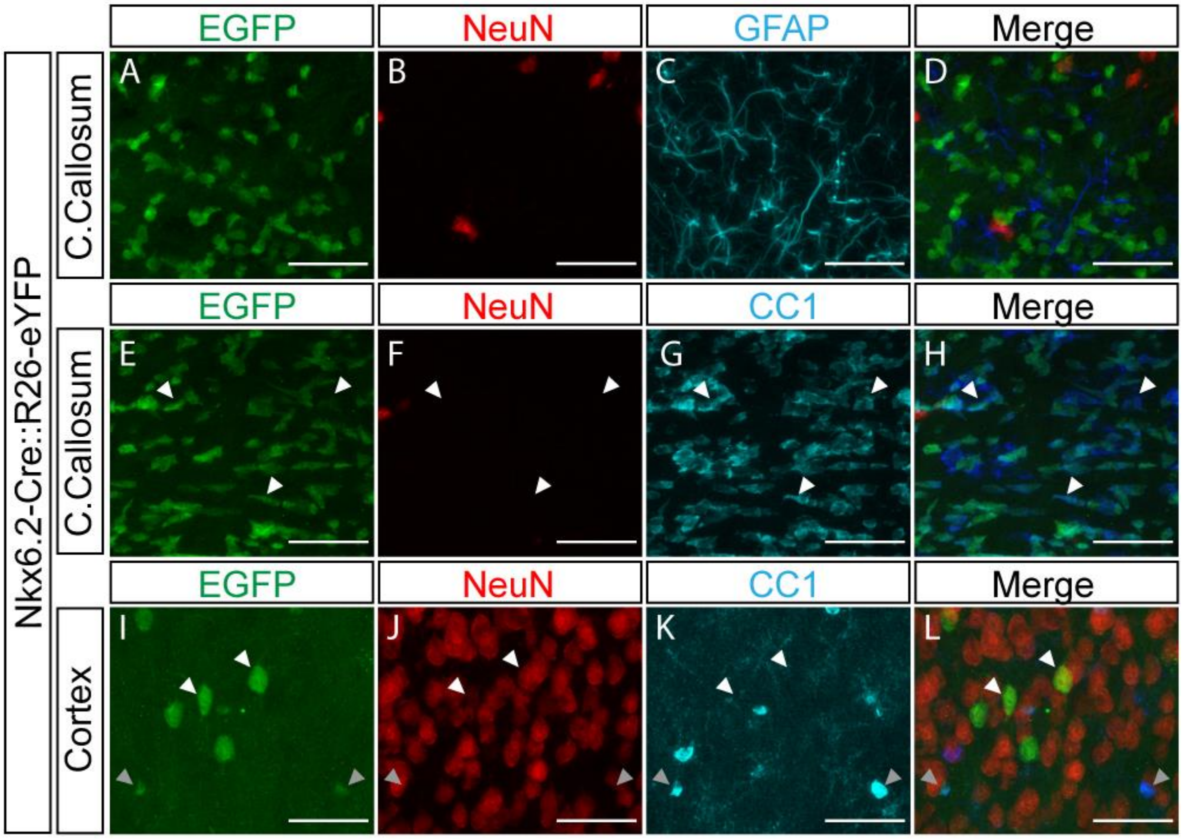

Supplementary Figure S3

Supplementary Figure S4. Nkx6.2 derived cortical interneurons infrequently express CGE or POA specific markers. (A-M) Immunofluorescences in coronal sections from Nkx6.2-Cre::R26-eYFP P30 mice for (A, D, G, J) EGFP, (B) VIP, (E) CR (H, L) RLN and (K) NPY. (C,F,I,M) Merged channels. (D-I) White arrowheads indicate interneurons derived from Nkx6.2 progenitors. (A-M) Gray arrowheads indicate interneurons in which there is no colocalization of markers analyzed. (N-Q) Quantification of (N) VIP, (O) CR, (P) RLN and (Q) RLN/NPY interneurons derived from Nkx6.2 progenitors respect to the total Nkx6.2 population (n=3-4). (R-U) Quantification of (R) VIP, (S) CR, (T) RLN and (U) RLN/NPY interneurons derived from Nkx6.2 progenitors from total VIP, CR, RLN and RLN/NPY population respectively (n=3-4). Images: 60X magnification from motor cortex; Bregma 0.5 mm. Scale bar: 50 µm. Values are presented as mean ± s.e.m.

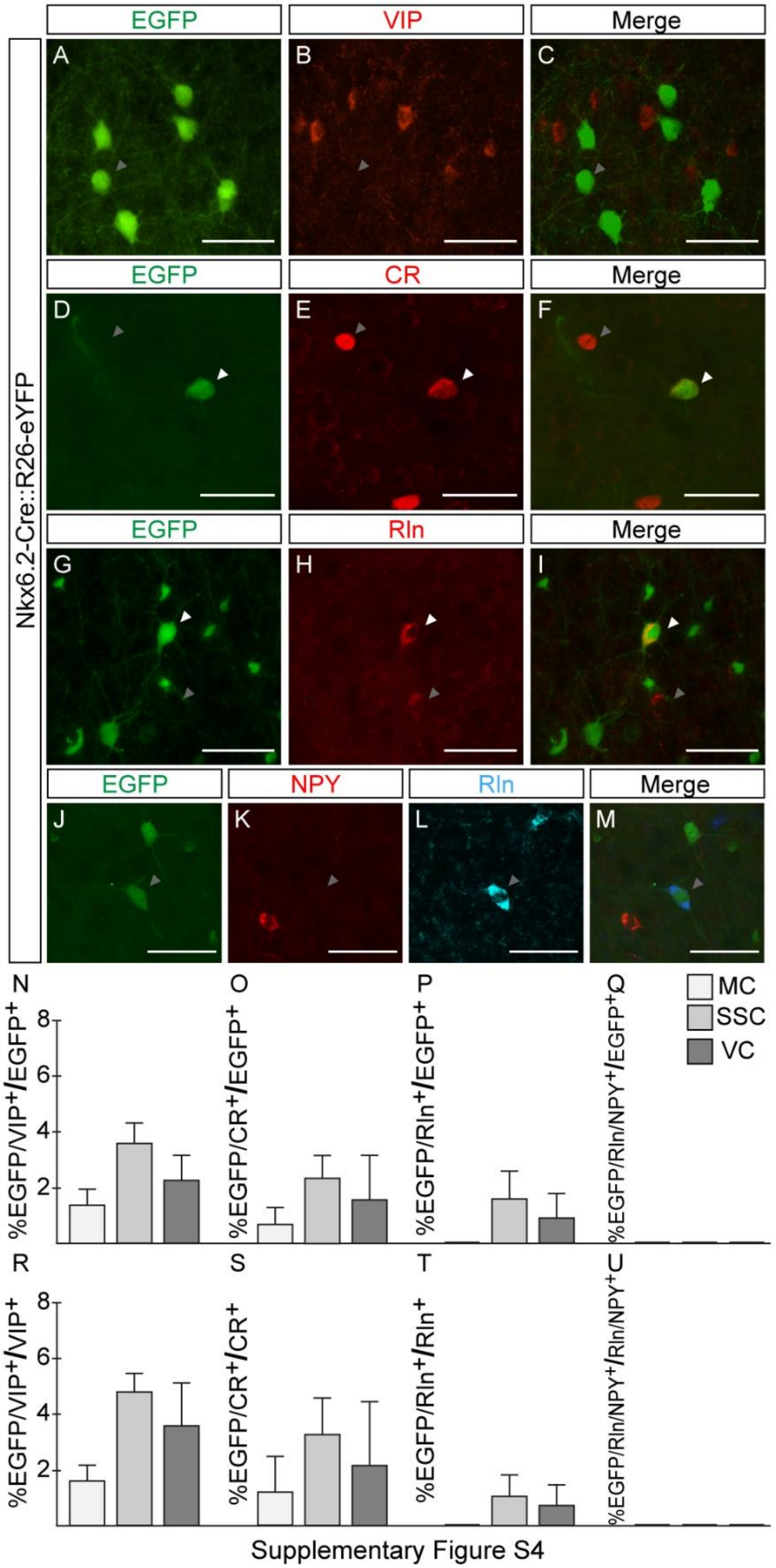

Supplementary Figure S4

Supplementary table S1:Individual behavioral data.

**Test**	**Analysis**	**WT**			**cKO**			**test**	**p**	**p value summary**
		mean	SEM	N		SEM	N			
Open field	Distance (mts)	94.3	7.05	14	87.8	7.52	14	unpaired t test	0.533	ns
Plus maze	Distance (mts)	17.34	1.01	15	15.52	1.42	14	unpaired t test	0.299	ns
Plus maze	% permanece time open	**10.98**	1.52	12	**15.87**	1.77	12	unpaired t test	0.046	*
Plus maze	% permanence time center	18.93	1.64	12	18.48	2.24	12	unpaired t test	0.87	ns
Plus maze	% permanece time closed	70.05	2.78	12	65.66	3.73	12	unpaired t test	0.362	ns
Y maze	spontaneous alternation	61.70	2.49	13	52.68	2.00	13	unpaired t test	0.0095	**
NOR	discrimination index	**6.457**	0.69	10	**1.091**	0.74	12	unpaired t test	<0.0001	***
Marble burying	buried marbles	**5.867**	0.95	15	**2.5**	0.69	14	Mann Whitney	0.0281	*
Nesting	% non used cotton	**77.55**	3.74	12	**87.85**	2.72	12	Mann Whitney	0.0141	*

The table shows individual values for the mean, SEM, number of animals used in the control (DRD2flox/flox) and conditional mutant (cKO, Nkx6.2 Cre-DRD2flox/flox) animal groups, the test used to analyze the data, the exact p value and the p value summary for each test performed during the study. Ns: not significant. * p<0.05; **p<0.01; ***p<0.0001. In bold, significantly different values.
